# Supplementary material for: Sharing refuges on arid islands: ecological and social influence on aggregation behaviour of wall geckos
Source: PeerJ. 2017 Jan 10;5:e2802. doi: 10.7717/peerj.2802 (PMC5228510; doi:10.7717/peerj.2802)
Supplement: Supplemental Information 1 [file peerj-05-2802-s001.docx]

**Appendix.** Abstract in Portuguese (official language of Cabo Verde).

**Resumo**

**Estado da arte.** Dois tipos de agregações são reconhecidos em lagartos: ecológicas e sociais, isto é, relacionadas com a atracção a um local ou a animais da mesma espécie, respectivamente. Como a maioria dos lagartos são territoriais, as agregações aumentam a probabilidade de interacções agressivas entre indivíduos, uma vez este comportamento é dependente da densidade.

**Métodos.** Realizámos trabalho de campo com a osga nocturna endémica de Cabo Verde *Tarentola substituta*. Para descrever a incidência, o tamanho e a composição das agregações, e estudar o efeito da densidade de osgas e refúgios e qualidade do refúgio na partilha de refúgios, foram amostrados 48 transectos e 40 quadrados de 10x10 m na ilha de São Vicente. Hipotetizamos assim que quando a densidade de animais e escassez de refúgios de elevada qualidade é mais alta, os lagartos tenham maior probabilidade de agregarem. Prevemos ainda que o padrão de tamanho e composição dos grupos seja consistente ao longo do ano (pares macho-fêmea com um único macho adulto) caso este comportamento seja seleccionado para evitar interacções agonísticas e pouca vantagem térmica para os indivíduos que se agregam.

**Resultados.** Apresentamos uma das primeiras evidências de agregação em osgas da família Phyllodactylidae. Constatámos que *T.* *substituta* forma agregações cerca de 30-40 % das vezes e que os refúgios são partilhados quase sempre por um par macho-fêmea, por vezes com um juvenil, provavelmente como mecanismo para evitar interacções agressivas. Observámos também que a partilha de refúgio é dependente da qualidade do refúgio, pois rochas médias-grandes (termicamente mais estáveis e seleccionadas positivamente) são partilhadas muito mais frequentemente do que as pequenas, mas independente do tamanho dos adultos. A partilha de refúgio está também directamente relacionada com a densidade de osgas e inversamente com a densidade de refúgios de elevada qualidade. Não encontramos nenhuma relação entre a temperatura do corpo das osgas e a partilha de refúgio quando controlamos o efeito da temperatura do ar e da rocha, sugerindo que estas não se juntam para melhorar a termorregulação.

**Discussão.** Os nossos resultados sugerem que a incidência da agregação neste ambiente hostil (as rochas podem atingir os 46ºC) é impulsionada principalmente por um factor ecológico (escassez de refúgios de elevada qualidade) e a composição intersexual por factores sociais (prevenção de interacções agressivas pelos machos e possível aumento do sucesso reprodutor do par). Este estudo lança alguma luz sobre o pouco explorado comportamento de agregação das osgas e outros estudos deverão seguir-se.
