# Supplementary material for: Sharing refuges on arid islands: ecological and social influence on aggregation behaviour of wall geckos
Source: PeerJ. 2017 Jan 10;5:e2802. doi: 10.7717/peerj.2802 (PMC5228510; doi:10.7717/peerj.2802)
Supplement: Table S1 — Total area (in squared meters) and percentage of cover of each habitat registered on São Vicente Island (Diniz & Matos, 1994), number of quadrats per habitat and total and average numbers (±squared deviation, SD) of adults, juveniles and total geckos found in quadrats of each habitat. [file peerj-05-2802-s002.docx]

**Supplementary Information**

**Table S1.** **Habitat and quadrats details.** Total area (in squared meters) and percentage of cover of each habitat registered on S. Vicente Island (Diniz & Matos, 1994), number of quadrats per habitat and total and average numbers (± standard deviation, SD) of adults, juveniles and total geckos found in quadrats of each habitat.

|  |  |  |  | **Adults** | | | **Juveniles** | | | **Total** | | |
| --- | --- | --- | --- | --- | --- | --- | --- | --- | --- | --- | --- | --- |
| **Habitat** | **Area** | **Cover** | **Quadrats** | **Sum** | **Average**  **± SD** | | **Sum** | **Average**  **± SD** | | **Sum** | **Average**  **± SD** | |
|  | **(km2)** | **(%)** |  |  |  |  |  |  |  |  |  |  |
| Saline | 2.4 | 1.1 | 0 | 0 | 0.0 |  | 0 | 0.0 |  | 0 | 0.0 |  |
| Sandy | 9.8 | 4.3 | 0 | 0 | 0.0 |  | 0 | 0.0 |  | 0 | 0.0 |  |
| Very arid flat | 72.3 | 32.1 | 11 | 8 | 0.7 | ±1.1 | 10 | 2.0 | ±2.4 | 18 | 1.6 | ±2.5 |
| Very arid and hilly | 29.9 | 13.2 | 11 | 37 | 3.4 | ±5.9 | 12 | 1.1 | ±1.2 | 49 | 4.5 | ±6.3 |
| Very arid and mountain | 80.7 | 35.8 | 8 | 12 | 1.5 | ±1.4 | 7 | 0.9 | ±0.8 | 19 | 2.4 | ±2.1 |
| Arid and hilly | 2.5 | 1.1 | 2 | 6 | 3.0 | ±4.2 | 0 | 0.0 |  | 6 | 3.0 | ±4.2 |
| Arid and mountain | 8.9 | 4.0 | 3 | 3 | 1.3 | ±1.5 | 3 | 0.7 | ±1.2 | 6 | 2.0 | ±1.0 |
| Semiarid and mountain | 6.7 | 3.0 | 3 | 6 | 2.0 | ±0.0 | 2 | 0.7 | ±0.6 | 8 | 2.7 | ±0.6 |
| Subhumid and mountain | 1.0 | 0.4 | 1 | 0 | 0.0 |  | 0 | 0.0 |  | 0 | 0.0 |  |
| Water lines and floodplains | 11.2 | 5.0 | 1 | 2 | 2.0 |  | 2 | 2.0 |  | 4 | 4.0 |  |
| Total | 225.4 | 100 | 40 | 74 | 1.9 | ±3.4 | 36 | 1.0 | ±1.3 | 110 | 2.8 | ±3.8 |
